# Post-translational regulation of the low-density lipoprotein receptor provides new targets for cholesterol regulation

**DOI:** 10.1042/BST20230918

**Published:** 2024-02-08

**Authors:** Harry Aldworth, Nigel M. Hooper

**Affiliations:** Division of Neuroscience, School of Biological Sciences, Faculty of Biology, Medicine and Health, University of Manchester, Manchester M13 9PT, U.K.

**Keywords:** low-density lipoprotein receptor, low-density lipoproteins, post-translational modification, proteolysis

## Abstract

The amount of the low-density lipoprotein receptor (LDLR) on the surface of hepatocytes is the primary determinant of plasma low-density lipoprotein (LDL)-cholesterol level. Although the synthesis and cellular trafficking of the LDLR have been well-documented, there is growing evidence of additional post-translational mechanisms that regulate or fine tune the surface availability of the LDLR, thus modulating its ability to bind and internalise LDL-cholesterol. Proprotein convertase subtilisin/kexin type 9 and the asialoglycoprotein receptor 1 both independently interact with the LDLR and direct it towards the lysosome for degradation. While ubiquitination by the E3 ligase inducible degrader of the LDLR also targets the receptor for lysosomal degradation, ubiquitination of the LDLR by a different E3 ligase, RNF130, redistributes the receptor away from the plasma membrane. The activity of the LDLR is also regulated by proteolysis. Proteolytic cleavage of the transmembrane region of the LDLR by γ-secretase destabilises the receptor, directing it to the lysosome for degradation. Shedding of the extracellular domain of the receptor by membrane-type 1 matrix metalloprotease and cleavage of the receptor in its LDL-binding domain by bone morphogenetic protein-1 reduces the ability of the LDLR to bind and internalise LDL-cholesterol at the cell surface. A better understanding of how the activity of the LDLR is regulated will not only unravel the complex biological mechanisms controlling LDL-cholesterol metabolism but also could help inform the development of alternative pharmacological intervention strategies for the treatment of hypercholesterolaemia.

## Introduction

Cholesterol is the principal sterol of all higher animals, with important roles in the structure of biological membranes and in cellular signalling pathways, both as a signalling molecule itself and as a precursor for other signalling molecules such as steroid hormones. In humans, cholesterol can be obtained either from *de novo* synthesis or via absorption from the diet, and is transported around the body via the bloodstream [1]. Cholesterol is hydrophobic and as such cannot freely dissolve in the blood plasma. Therefore, cholesterol is packaged along with other lipids and proteins into lipoprotein particles for transport around the body, with low-density lipoprotein (LDL) being the primary carrier of cholesterol in humans [2].

Appropriate regulation of cholesterol homeostasis is fundamental for human health, with dysregulation of cholesterol homeostasis being implicated in a range of diseases, such as familial hypercholesterolaemia, Alzheimer's disease and most notably cardiovascular disease (CVD) [3–5]. CVD is the leading cause of death in the world [6], and elevated blood LDL-cholesterol (hypercholesterolaemia) is a major risk factor for this because LDL-cholesterol promotes the formation of atherosclerotic plaques on the walls of blood vessels, narrowing and eventually completely occluding blood flow [7]. As a result, the development of treatments to counteract hypercholesterolaemia is important in the prevention of the potentially fatal consequences, including heart attacks and strokes. The most common current treatments for hypercholesterolaemia target three main mechanisms or proteins in cholesterol homeostasis: (i) *de novo* cholesterol synthesis using statins that inhibit 3-hydroxy-3-methylglutaryl (HMG) CoA reductase (the rate-limiting enzyme in the cholesterol synthetic pathway) [8]; (ii) cholesterol absorption in the gut using ezetimibe to block the receptor that binds and internalises bile-solubilised cholesterol [9]; and (iii) proprotein convertase subtilisin/kexin type 9 (PCSK9)-mediated regulation of the low-density lipoprotein receptor (LDLR) using monoclonal antibodies or short interfering RNAs against PCSK9 to block the action or reduce the expression of PCSK9, respectively [10,11]. Despite their clinical success in the majority of individuals, these treatments can be insufficient in restoring plasma cholesterol levels to within a normal physiological range [12]. In addition, in some patients, these treatments can cause a rebound in plasma cholesterol following the withdrawal of the drug(s) to higher than that before treatment [13], or increase the risk of developing other conditions such as type 2 diabetes [14]. Thus, there is still a need to develop alternative drugs to reduce LDL-cholesterol.

**Figure 1.**
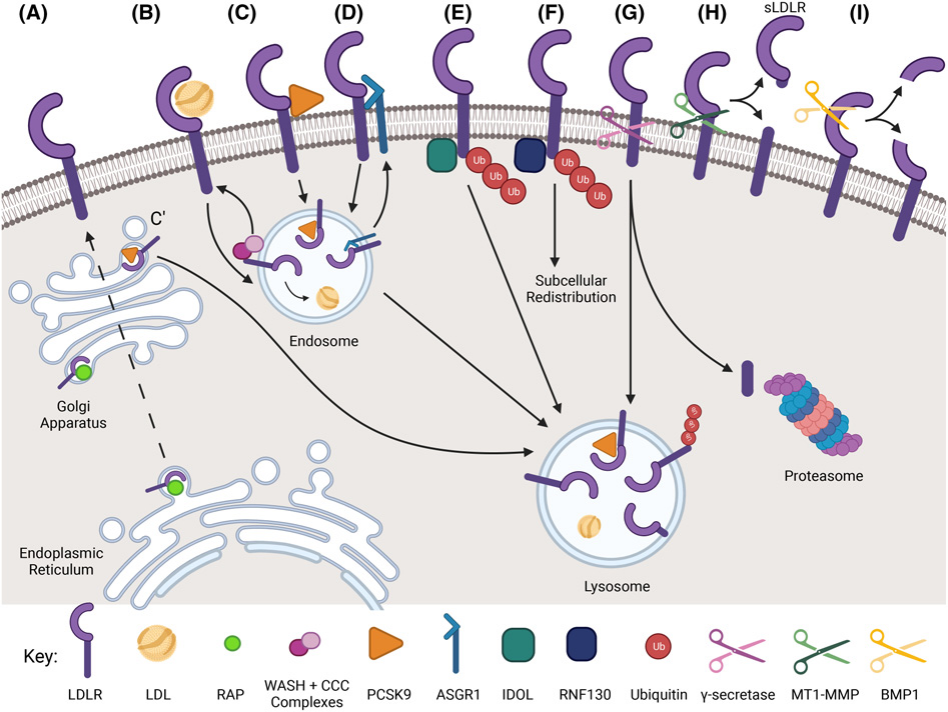
Post-translational regulation of the LDLR. The range of currently identified mechanisms by which the surface availability of the LDLR is controlled is indicated. (**A**) Synthesis of the LDLR and its trafficking to the cell surface with the chaperone protein RAP. (**B**) Internalisation of LDL-bound LDLR and recycling of the receptor back to the plasma membrane. (**C**) Extracellular binding of PCSK9 to the LDLR and subsequent targeting to the lysosome for degradation. (**C**’) Targeting of the LDLR to the lysosome by PSCK9 at the trans-Golgi. (**D**) Endocytosis and targeting of the LDLR to the lysosome for degradation by ASGR1. (**E**) Polyubiquitination of the LDLR by IDOL and targeting to the lysosome for degradation. (**F**) Polyubiquitination of the LDLR by RNF130, redistributing the receptor to the endosome, away from the plasma membrane. (**G**) Cleavage of the LDLR by γ-secretase, with the cleaved cytosolic domain being degraded by the proteasome, and the remaining membrane-bound receptor being targeted to the lysosome for degradation. (**H**) Extracellular cleavage of the LDLR by MT1-MMP, releasing soluble LDLR (sLDLR) into the extracellular space. (**I**) Cleavage of the LDLR by BMP1 releasing an N-terminal fragment into the extracellular space and retaining a C-terminal fragment in the plasma membrane. Note: there is no specific rationale for the order of the post-translational modifications to LDLR in the figure. Created with Biorender.com.

The LDLR is the primary regulator of plasma LDL-cholesterol levels, with the majority of this regulation (∼70%) occurring at the liver, although the LDLR is ubiquitously expressed in all tissues [15]. The LDLR is a modular single-pass transmembrane glycoprotein that is present at the plasma membrane where it binds and internalises extracellular LDL-cholesterol. The availability and activity of the LDLR at the cell surface, particularly of hepatocytes, is a key mechanism in the regulation of plasma LDL-cholesterol levels [15]. Although the synthesis and cellular trafficking of the LDLR have been well-documented, there is growing evidence of multiple post-transcriptional and post-translational mechanisms that regulate or fine tune the cell surface availability of the receptor, thus modulating its ability to bind and internalise LDL-cholesterol. A better understanding of how the LDLR is regulated will not only unravel the complex biological mechanisms controlling LDL-cholesterol metabolism but also could help inform the development of next-generation pharmacological intervention strategies for the treatment of hypercholesterolaemia. Below we briefly mention some of the transcriptional and post-transcriptional mechanisms that regulate the expression of the LDLR, but the focus of this article is on the growing number of post-translational mechanisms, many only identified in recent years, that regulate the amount of active LDLR present at the plasma membrane (summarised in Figure 1) and how these mechanisms are providing new targets for regulation of LDL-cholesterol.

## LDLR regulation

**Figure 2.**
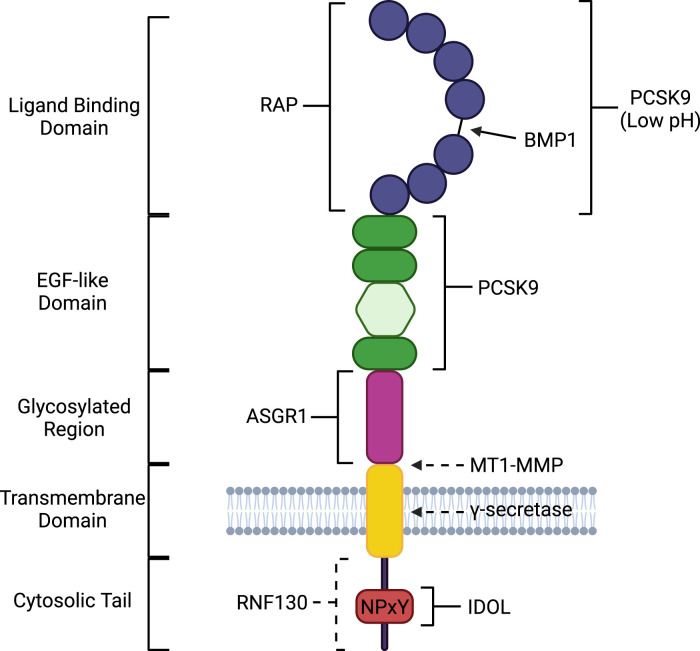
Structure of the LDLR shows where other proteins bind or cleave to regulate its function. Schematic of the domain structure of the LDLR with the ligand-binding (LA) repeats in blue, the EGF-like domains in dark green, the β-propellor domain in light green, the glycosylated juxtamembrane region in pink, the transmembrane region in yellow and the cytosolic domain containing the NPxY motif (in red). The binding sites (in square brackets) for RAP, PCSK9, ASGR1, IDOL and RNF130 are shown, along with the positions (arrows) in the receptor that are cleaved by BMP1, MT1-MMP and γ-secretase. Experimentally confirmed binding and cleavage sites are shown with solid lines, and proposed binding and cleavage sites are shown with dashed lines. Created with Biorender.com.

The domain structure of the LDLR, as well as sites of interaction and cleavage by other proteins (details of which are presented later), are shown in Figure 2. The LDLR binds LDL via an extracellular ligand binding domain composed of 7 cysteine-rich LDLR type A (LA) repeats, each separated by a short linker of a few amino acids [16] (Figure 2). This ligand binding domain recognises the protein component of LDL particles, apolipoprotein B100, and, to a lower affinity, apolipoprotein E which is present in very low-density lipoprotein particles and chylomicrons [17,18]. Glycosylation (both N-linked and O-linked) within the ligand binding domain has been shown to enhance the ligand binding affinity of the LDLR [19–21]. Once bound to the LDLR, the receptor–lipoprotein complex is internalised and transported through the endosomal system [22] (Figure 1B). In the low pH environment of the endosome, the LDLR undergoes a conformational change whereby its epidermal growth factor (EGF)-like repeat domains bind to the ligand binding domain, displacing the LDL from the receptor [23]. The free LDL is transported to the lysosome where it is degraded to release the contained cholesterol and other lipids [24], while the receptor has two possible routes following dissociation (Figure 1B). It too can be transported to the lysosome for degradation or it can be recycled to the plasma membrane to bind and internalise more LDL, with the fate of the LDLR depending on the amount of cholesterol present in the cell. Efficient LDLR recycling requires the Wiskott–Aldrich syndrome protein and SCAR homologue (WASH) and the COMMD/CCDC22/CCDC93 (CCC) complex (Figure 1B) [25]. A coding variant in CCDC93 increases the stability of the protein and is associated with lower LDL-cholesterol, lower risk of myocardial infarction and of CVD mortality [26], suggesting that endosomal trafficking and/or recycling of the LDLR could be a target for pharmaceutical intervention.

### Transcriptional regulation

The expression of the LDLR is under the control of the transcription factor sterol response element binding protein 2 (SREBP2), which is regulated by cellular cholesterol status via interaction with a cholesterol-sensing protein, SREBP cleavage-activating protein (SCAP) [27]. When cellular cholesterol is elevated, the SCAP–SREBP2 complex cannot be transported to the Golgi and so SREBP2 is sequestered at the membrane of the endoplasmic reticulum (ER) as an inactive precursor [28,29]. When cellular cholesterol is depleted, the SCAP–SREBP2 complex is transported to the Golgi where SREBP2 is activated and released into the cytosol from where it translocates to the nucleus and up-regulates the expression of target genes, including *LDLR*. The newly synthesised LDLR is trafficked to the plasma membrane where it increases cellular uptake of LDL-cholesterol and restores cholesterol homeostasis. At the post-transcriptional level, LDLR mRNA is regulated via several mechanisms including multiple microRNAs [30], alternative splicing to produce non-functional LDLR protein [31], and regulation of LDLR mRNA decay [32].

### ER quality control and RAP chaperone

An important regulatory mechanism for the LDLR in the secretory trafficking pathway is ER quality control. Mutations have been identified in the LDLR that cause retention of the LDLR at the ER, ultimately leading to ER-associated decay of the protein. These mutations can disrupt the folding of the LDLR, such as abnormal *N*-glycosylation that is required for normal folding [33], or prevent insertion of the LDLR into the ER membrane [34]. As with all proteins, ER chaperone proteins are important for correct folding and ER exit, with the chaperone receptor-associated protein (RAP) being especially important for the LDLR. RAP binds to the ligand binding domain of the LDLR (Figure 2) in the ER [35] to promote transport along the secretory pathway towards the plasma membrane (Figure 1A), with this association between RAP and the LDLR also reducing the affinity of the receptor for LDL [36]. In addition, the binding of RAP to the LDLR can influence the activity of other processing mechanisms that regulate the LDLR, such as blocking LDLR proteolytic cleavage by bone morphogenetic protein-1 (BMP1) [37] (see below). Thus, RAP has an important role in the regulation of LDLR surface availability via chaperoning the receptor as it is trafficked along the secretory pathway and by modulating the ability of other proteins to interact with the receptor.

### PCSK9

A major mechanism that modulates the cell surface level of the LDLR is its targeted lysosomal degradation by PCSK9. The secreted PCSK9 binds the EGF-like domain in the extracellular region of the LDLR (Figure 2) and promotes the internalisation of the receptor via clathrin-mediated endocytosis [38] (Figure 1C). Like the LDLR, PCSK9 is under the regulation of SREBP2 and its expression is, therefore, also responsive to the cellular cholesterol level [27]. Following PCSK9-mediated internalisation, the LDLR–PCSK9 complex is delivered to the endosome. In the low pH environment of the endosome, PCSK9 becomes more strongly bound to the LDLR through additional interaction with the ligand binding domain of the receptor (Figure 2). This contrasts with the LDLR–LDL complex which dissociates in the acidic endosome. By remaining bound to the LDLR, PCSK9 prevents the conformational change of the LDLR that is required for its recycling back to the plasma membrane, therefore, inhibiting this recycling [38]. Instead, the PCSK9–LDLR complex is directed to the lysosome where both proteins are degraded (Figure 1C), thereby reducing the amount of LDLR in the cell [39].

In addition, PCSK9 can bind newly synthesised LDLR at the trans-Golgi network and target it directly to the lysosome before it has reached the plasma membrane (Figure 1C), with disruption of this intracellular transport between the trans-Golgi network and lysosome resulting in an increase in cell surface LDLR [40]. This provides two separate routes by which PCSK9 can regulate the surface availability of the LDLR. Interestingly, PCSK9 has also been shown to bind the protein component of LDL particles, apolipoprotein B100 [41]. This can prevent interaction between PCSK9 and LDLR, and subsequent targeted degradation, by sequestering PCSK9 in the blood plasma, providing a secondary regulatory mechanism for PCSK9 to enhance cholesterol uptake when plasma LDL-cholesterol levels are elevated, thus helping to maintain cholesterol homeostasis. As mentioned above treatments targeting PCSK9 are already in clinical use for hypercholesterolemia.

### ASGR1

Another protein that promotes lysosomal degradation of the LDLR is asialoglycoprotein receptor 1 (ASGR1). ASGR1 is the major subunit of the dimeric transmembrane asialoglycoprotein receptor primarily expressed in hepatocytes, which binds glycoproteins and promotes their endocytosis and subsequent lysosomal degradation. As a glycoprotein also highly expressed in hepatocytes, the LDLR is targeted by ASGR1 (Figure 1D). Knockdown of ASGR1 increased the total amount of LDLR in the cell and increased the uptake of LDL, showing that ASGR1 normally negatively regulates LDLR availability and, as a result, LDL uptake [42]. Loss-of-function mutations in the *ASGR1* gene are associated with decreased levels of non-high-density lipoprotein cholesterol and triglycerides in the blood plasma, resulting in a reduction in the risk of atherosclerosis and CVD [43]. Knockout of *ASGR1* had the same effect [44]. Together, this shows the role of ASGR1 in the post-translational regulation of the LDLR, and suggests that silencing of ASGR1 may provide a novel approach to reduce plasma LDL-cholesterol levels and thus protect against the formation and progression of atherosclerosis.

### IDOL

Ubiquitination of the LDLR by the inducible degrader of the LDLR (IDOL) also targets the receptor to the lysosome for degradation (Figure 1E). IDOL is an E3 ubiquitin ligase that polyubiquitinates LDLR in complex with the E2 ubiquitin-conjugating enzyme UBE2D [45]. IDOL is recruited to the NPxY motif in the intracellular domain of the LDLR (Figure 2) via its FERM domain and catalyses polyubiquitination of the LDLR cytosolic tail [46]. This polyubiquitination targets the LDLR to the lysosome via selective sorting into multivesicular bodies that are transported to, and fuse with, the lysosome. This protein sorting and lysosomal targeting requires proteins from the epsin-family and ESCRT complexes [47]. Some evidence suggests that deubiquitination of the LDLR following IDOL-mediated polyubiquitination also plays an important role in this targeted LDLR degradation, with knockdown of USP8, a deubiquitinating enzyme, causing an accumulation of polyubiquitinated LDLR species in IDOL-expressing cells. This suggests that polyubiquitination of the LDLR alone is not sufficient for targeted degradation but rather initial polyubiquitination with subsequent deubiquitination is required to facilitate LDLR degradation by this pathway [48]. However, there is also contradictory evidence suggesting that the action of USP8 may prevent IDOL-mediated LDLR degradation, with co-expression of IDOL and USP8 resulting in an increase in total cellular LDLR [47]. As such, the exact mechanics of the IDOL–USP8 ubiquitination system in the control of LDLR levels remain unclear, but what is clear is that IDOL decreases the surface levels of the LDLR, thereby decreasing cellular uptake of LDL-cholesterol.

Like the expression of the LDLR and PCSK9, the expression of IDOL is also sensitive to the cholesterol status of the cell, although not via SREBP2. Instead, IDOL expression is regulated by the liver x receptor nuclear receptor family of proteins (LxRs). LxRs are responsive to the presence of oxysterol, an intermediate in cholesterol metabolism, and provide a secondary measure of the cholesterol status of the cell. In their resting state, LxRs are bound to a corepressor protein that retains them in a sequestered, inactive state within the nucleus. When cellular cholesterol levels, and therefore oxysterol levels, are elevated, the oxysterol binds the LxRs, causing a conformational change that promotes the exchange of the corepressor for a co-activator, facilitating the transcriptional activation of target genes such as *IDOL* [49]. This is in contrast with SREBP2 which is activated in response to reduced cellular cholesterol. Interestingly, the LxR–IDOL axis differentially regulates plasma LDL levels in primates and mice [50]. In mice, an LxR agonist induced *Idol* transcript levels in peripheral tissues but not in the liver and did not change plasma LDL levels. Whereas LxR activation in monkeys induced hepatic *IDOL* expression, reduced LDLR levels and raised plasma LDL, supporting IDOL inhibition as a potential strategy for lowering LDL-cholesterol in humans.

### RNF130

IDOL is not the only ubiquitination mechanism that regulates LDLR surface levels. RNF130, also known as GOLIATH, another E3 ligase, has recently been implicated in LDLR regulation [51]. RNF130 polyubiquitinates the LDLR but the functional consequence of this ubiquitination is different from that catalysed by IDOL. Although RNF130 decreases total LDLR levels showing its polyubiquitination promotes targeted degradation of the receptor, the more significant effect of RNF130 activity against the LDLR is the subcellular redistribution of the receptor (Figure 1F). Polyubiquitination of the LDLR by RNF130 primarily functions to redistribute the receptor away from the cell surface, reducing the ability of the receptor to internalise LDL and providing a reversible mechanism for the regulation of LDLR activity [51]. Disruption of *Rnf130* using antisense oligonucleotides *in vivo* in mice resulted in increased hepatic LDLR abundance and availability and decreased plasma LDL-cholesterol levels [51], indicating that the RNF130-LDLR pathway could be targeted to enhance plasma LDL-cholesterol clearance.

### γ-secretase

Targeted lysosomal degradation of the LDLR in hepatocytes has also been observed following proteolytic cleavage of the receptor by γ-secretase [52] (Figure 1G). γ-secretase is a protease complex composed of four transmembrane protein subunits (nicastrin, presenilin-1 or 2, PEN2 and Aph-1) which is most typically associated with cleavage of the amyloid precursor protein (APP) in the production of the amyloid-β peptide, the primary component of amyloid plaques seen in the brains of Alzheimer's patients [53]. The nicastrin subunit of γ-secretase binds to the LDLR and recruits the rest of the γ-secretase complex, resulting in the cleavage of the LDLR (γ-secretase normally cleaves substrates within its transmembrane domain so this is likely also the case for the LDLR (Figure 2), though the exact cleavage site has yet to be experimentally confirmed). The released intracellular cytosolic domain of LDLR is degraded by the proteasome (Figure 1G). The lack of its cytosolic domain destabilises the LDLR and targets what is left of the receptor to the lysosome for degradation [52]. Normally, the γ-secretase complex is autoinhibited by the nicastrin subunit, where nicastrin binds and blocks the active site of the catalytic presenilin subunit, inhibiting its activity. To remove this autoinhibition, most substrates are cleaved extracellularly, leaving a short extracellular tail on the transmembrane region which nicastrin binds to and which then exposes the γ-secretase active site, such as in APP which is initially cleaved extracellularly by β-secretase [54]. However, γ-secretase-mediated cleavage of the LDLR has been shown to occur on the uncleaved full-length receptor, although there is the possibility that the receptor may be cleaved extracellularly prior to γ-secretase cleavage [52] and in this respect, the action of membrane-type 1 matrix metalloproteinase (MT1-MMP) should be considered (see below). Liver-selective γ-secretase inhibition ameliorated diet-induced dyslipidaemia in mice [55], indicating that targeting the γ-secretase cleavage of the LDLR could provide an alternative therapeutic approach to treat hypercholesterolemia.

### MT1-MMP

In addition to the proteolytic cleavage of the LDLR by γ-secretase, two zinc metalloproteases also cleave the receptor, MT1-MMP and BMP1. MT1-MMP cleaves the LDLR adjacent to the plasma membrane (Figures 1H and 2) [56]. MT1-MMP is a transmembrane protease that has major and well-established functions in the remodelling of extracellular matrix proteins [57]. Recent research has identified MT1-MMP as a regulator of LDLR cell surface availability, with knockout of hepatic MT1-MMP leading to an increase in cellular LDLR levels and a reduction in plasma cholesterol, and overexpression of MT1-MMP decreasing total LDLR levels, increasing plasma cholesterol levels and increasing atherosclerotic lesions [58]. The extracellular cleavage of the LDLR catalysed by MT1-MMP releases almost the entire ectodomain intact into the extracellular space as a soluble form of the receptor (sLDLR) (Figure 1H). The sLDLR retains the complete ligand binding domain and is still able to bind lipoprotein particles, potentially preventing their binding to the intact cell surface form of the receptor [58]. Therefore, the MT1-MMP mediated cleavage of the LDLR provides a two-fold regulatory mechanism; reducing the amount of full-length LDLR at the cell surface, and generating a soluble form of the receptor that can sequester the LDL in the extracellular space, preventing its interaction with any uncleaved receptor on the cell surface. MT1-MMP is highly expressed in various types of cancer cells and promotes cancer metastasis and angiogenesis [57,59]. Thus, inhibition of MT1-MMP is a promising therapeutic target to not only increase hepatic LDLR levels and lower plasma LDL-cholesterol, but also to reduce the risk of cancer metastasis and invasion [58].

### BMP1

Another example of the regulation of LDLR surface availability via proteolysis is through the action of BMP1. Like MT1-MMP, BMP1 is a metalloprotease with major roles in remodelling of extracellular matrix proteins, but BMP1 is a secreted protease rather than transmembrane [60]. The cleavage of the LDLR by BMP1 is also markedly different from MT1-MMP. BMP1 cleaves the LDLR within the ligand binding domain (Figure 2), between LA repeats 4 and 5 where the linker between the repeats is slightly larger, at 12 amino acids rather than 4–5 amino acids as in the linkers between the other LA repeats [37]. The BMP1-mediated cleavage of LDLR releases a small N-terminal fragment of 36–40 kDa and retains a C-terminal fragment of 120 kDa in the plasma membrane (Figure 1I) [37,61]. For decades, the existence of this 120 kDa LDLR species has been known but was attributed to an immature form of the LDLR prior to its complete glycosylation, hence the lower molecular mass than that observed for mature LDLR (which is 160 kDa) [62]. However, recent research has shown that this 120 kDa species is in fact primarily a BMP1 cleavage product present at the cell surface and not an intracellular immature form of the receptor [63]. BMP1 cleavage within the ligand binding domain of the LDLR significantly reduces the affinity of the receptor for LDL, decreasing the ability of the LDLR to bind and internalise LDL [37].

The site of cleavage in human LDLR by BMP1 is highly specific, between residues Gly192 and Asp193, with the Asp residue being crucial for cleavage [37,61,63]. In murine LDLR, this Asp residue is replaced with a Val and murine LDLR is not cleaved by BMP1 [37], so studies in mice expressing wild-type LDLR would not reveal whether cleavage of the receptor by BMP1 has any impact on LDL-cholesterol metabolism. However, there is evidence that BMP1 is involved in cleaving the LDLR in human primary hepatocytes and in human liver and adrenal samples [37,61,63], and the 36–40 kDa N-terminal fragment is present in human urine, likely due to clearance from the blood plasma by the kidneys [61]. Both 14-mer synthetic peptides and antibodies directed against the linker region between LA repeats 4 and 5 prevented cleavage of LDLR by BMP1, providing proof of principle that blocking the action of BMP1 on LDLR could represent a novel approach to reducing plasma LDL-cholesterol [64].

## Conclusion and future perspectives

Cholesterol synthesis, cellular uptake and degradation are tightly regulated. Proper cholesterol homeostasis is crucial for human health, with elevated plasma cholesterol implicated in a range of major diseases, and as such, effective treatment of hypercholesterolemia is key to reduce the risk and incidence of CVD, dementia and stroke. Despite the success of current therapeutics that are prescribed for hypercholesterolemia, there is the ongoing need to provide better control of plasma cholesterol levels and to identify alternative therapeutic targets.

The LDLR is a major point of regulation in cholesterol homeostasis. The extensive array of post-translational processing mechanisms that regulate the availability of the LDLR at the hepatocyte plasma membrane provides multiple potential points for pharmacological intervention. Indeed, as mentioned above, there are already drugs developed that target the PCSK9-mediated degradation of the LDLR, but this is just one of many proteins that are involved in the post-translational processing of the receptor that could be utilised to develop new, additional therapeutics for the treatment of cholesterol dysregulation.

Many of the proteins that act on the LDLR are promiscuous, having multiple other substrates that may pose difficulties for selective drug development. Also, regulation of LDLR activity differs between primates and rodents (IDOL and BMP1 only act on primate LDLR). Thus, further research into the post-translational mechanisms that regulate the receptor is necessary to fully understand the complexity of LDLR regulation in humans and to identify how such mechanisms could be successfully targeted to increase LDLR surface availability without adverse effects on other critical biological processes, ultimately leading to the development of new therapeutic strategies to treat hypercholesterolemia and its associated clinical consequences.

## Data Availability

All supporting data are included within the main article.

## Open Access

Open access for this article was enabled by the participation of University of Manchester in an all-inclusive *Read & Publish* agreement with Portland Press and the Biochemical Society under a transformative agreement with JISC.

## Abbreviations

APP: amyloid precursor protein
ASGR1: asialoglycoprotein receptor 1
BMP1: bone morphogenetic protein-1
CVD: cardiovascular disease
EGF: epidermal growth factor
ER: endoplasmic reticulum
IDOL: inducible degrader of the LDLR
LA: LDLR type A
LDL: low-density lipoprotein
LDLR: low-density lipoprotein receptor
LxR: liver × receptor
MT1-MMP: membrane-type 1 matrix metalloprotease
PCSK9: proprotein convertase subtilisin/kexin type 9
RAP: receptor-associated protein
sLDLR: soluble LDLR
SREBP2: sterol response element binding protein 2
